# Supplementary material for: Electrowetting on liquid-infused film (EWOLF): Complete reversibility and controlled droplet oscillation suppression for fast optical imaging
Source: Sci Rep. 2014 Oct 30;4:6846. doi: 10.1038/srep06846 (PMC4213809; doi:10.1038/srep06846)
Supplement: Supplementary Information — Supporting information [file srep06846-s1.pdf]

Supporting information

Electrowetting on liquid-infused film (EWOLF): Complete reversibility and controlled droplet oscillation suppression for fast optical imaging

Chonglei Hao[1], Yahua Liu[1], Xuemei Chen[1], Yuncheng He[2], Qiusheng Li[2], K.Y. Li[1],
Zuankai Wang[1,*]

[1]Department of Mechanical and Biomedical Engineering, City University of Hong Kong, Hong Kong, China
[2]Department of Civil and Architectural Engineering, City University of Hong Kong, Hong Kong, China

*Corresponding author. E-mail: zuanwang@cityu.edu.hk

Drop stable configuration on lubricant infused film.

To determine the stable configuration for the water droplet placed on lubricant infused film, we first measured the contact angle of oil drop (FC-70 and Krytox 103) on the smooth Teflon coating surfaces under air ($\theta_{os(a)}$) and the water environment ($\theta_{os(w)}$), where subscripts o, s, a, and w represent for oil, solid, air and water, respectively[1,2]. Fig. S1 shows the time evolution of FC-70 drop spreading in the air **(a)** and water **(b)** environments. It is obvious that the apparent contact angle in the air ($\theta_{os(a)}$) and water ($\theta_{os(w)}$) environment is close to 0°, suggesting that the oil can preferentially wet the Teflon matrix and the deposited droplet is floating on the oil-infused film. We further calculated the spreading parameter $S = \gamma_{wa} - (\gamma_{wo} + \gamma_{oa})$, where γ_{wa}, γ_{wo}, and γ_{oa} are the surface tensions of water-air, water-oil, and oil-air interfaces, respectively. As listed in Table S1, the spreading parameters of two oils on water are both larger than 0, revealing that a thin oil film will cloak the water drop at zero voltage, as shown in Fig. S2.

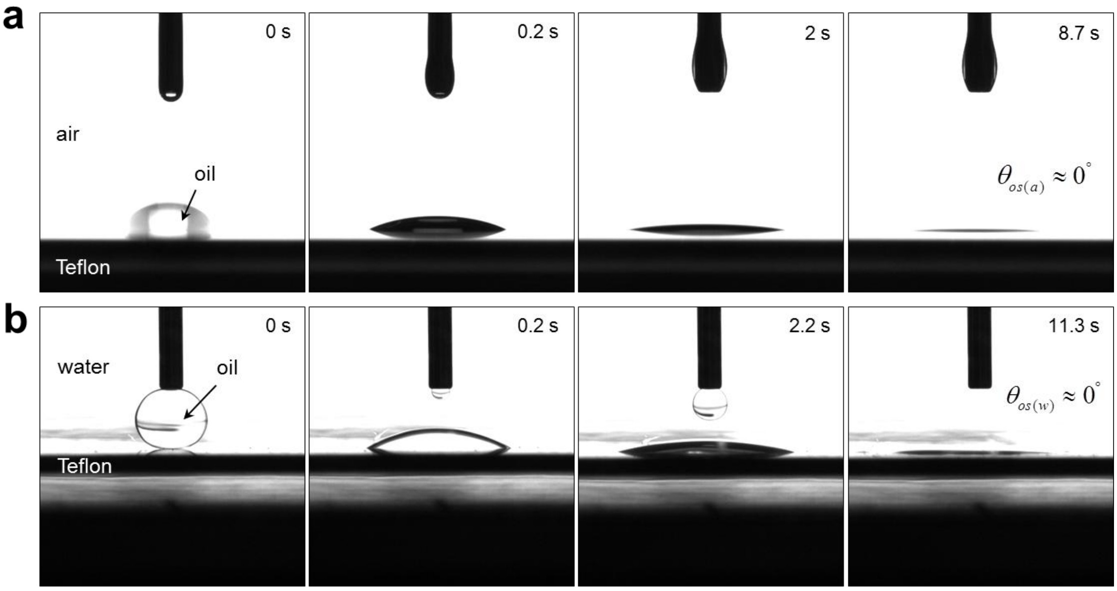

Figure S1: Time evolution of FC-70 drop morphology placed on smooth Teflon coating surface in the air **(a)** and water **(b)** environment, respectively. Both of the final contact angles, $\theta_{os(a)}$ and $\theta_{os(w)}$, were nearly 0 °.

Table S1. Physical properties of oil and the calculated spreading parameter.

oil	Surface tension, γ_{oa}, mN/m	Surface tension γ_{wo}, mN/m	Spreading parameter, S, mN/m
FC-70	16.2	51.4 ±1	4.4 ±1
Krytox103	18.4	47 ±1	6.6 ±1

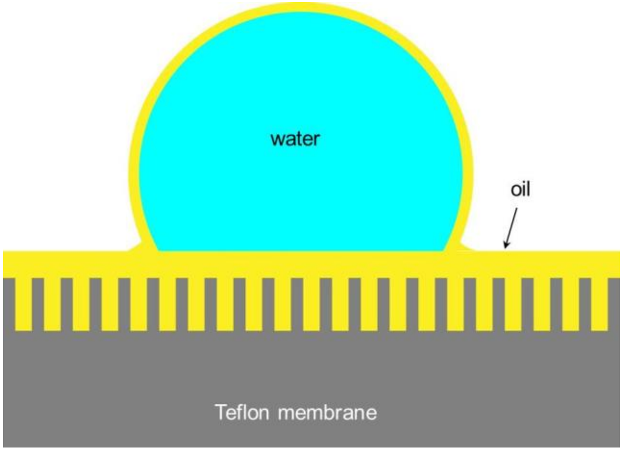

Figure S2: Schematic diagram of a water drop placed on the lubricant infused film demonstrates the floating state of water drop.

References

1. Smith, J. D., et al. Droplet mobility on lubricant-impregnated surfaces. *Soft Matter* **9**, 1772-1780 (2013).
2. Wong, T. S., et al. Bioinspired self-repairing slippery surfaces with pressure-stable omniphobicity. *Nature* **477**, 443-447 (2011).
